# Machine learning techniques for forecasting agricultural prices: A case of brinjal in Odisha, India

**DOI:** 10.1371/journal.pone.0270553

**Published:** 2022-07-06

**Authors:** Ranjit Kumar Paul, Md. Yeasin, Pramod Kumar, Prabhakar Kumar, M. Balasubramanian, H. S. Roy, A. K. Paul, Ajit Gupta

**Affiliations:** 1 ICAR-Indian Agricultural Statistics Research Institute, New Delhi, India; 2 ICAR-Indian Agricultural Research Institute, New Delhi, India; The Bucharest University of Economic Studies, ROMANIA

## Abstract

**Background:**

Price forecasting of perishable crop like vegetables has importance implications to the farmers, traders as well as consumers. Timely and accurate forecast of the price helps the farmers switch between the alternative nearby markets to sale their produce and getting good prices. The farmers can use the information to make choices around the timing of marketing. For forecasting price of agricultural commodities, several statistical models have been applied in past but those models have their own limitations in terms of assumptions.

**Methods:**

In recent times, Machine Learning (ML) techniques have been much successful in modeling time series data. Though, numerous empirical studies have shown that ML approaches outperform time series models in forecasting time series, but their application in forecasting vegetables prices in India is scared. In the present investigation, an attempt has been made to explore efficient ML algorithms e.g. Generalized Neural Network (GRNN), Support Vector Regression (SVR), Random Forest (RF) and Gradient Boosting Machine (GBM) for forecasting wholesale price of Brinjal in seventeen major markets of Odisha, India.

**Results:**

An empirical comparison of the predictive accuracies of different models with that of the usual stochastic model i.e. Autoregressive integrated moving average (ARIMA) model is carried out and it is observed that ML techniques particularly GRNN performs better in most of the cases. The superiority of the models is established by means of Model Confidence Set (MCS), and other accuracy measures such as Mean Error (ME), Root Mean Square Error (RMSE), Mean Absolute Error (MAE) and Mean Absolute Prediction Error (MAPE). To this end, Diebold-Mariano test is performed to test for the significant differences in predictive accuracy of different models.

**Conclusions:**

Among the machine learning techniques, GRNN performs better in all the seventeen markets as compared to other techniques. RF performs at par with GRNN in four markets. The accuracies of other techniques such as SVR, GBM and ARIMA are not up to the mark.

## Introduction

**Fig 1:**
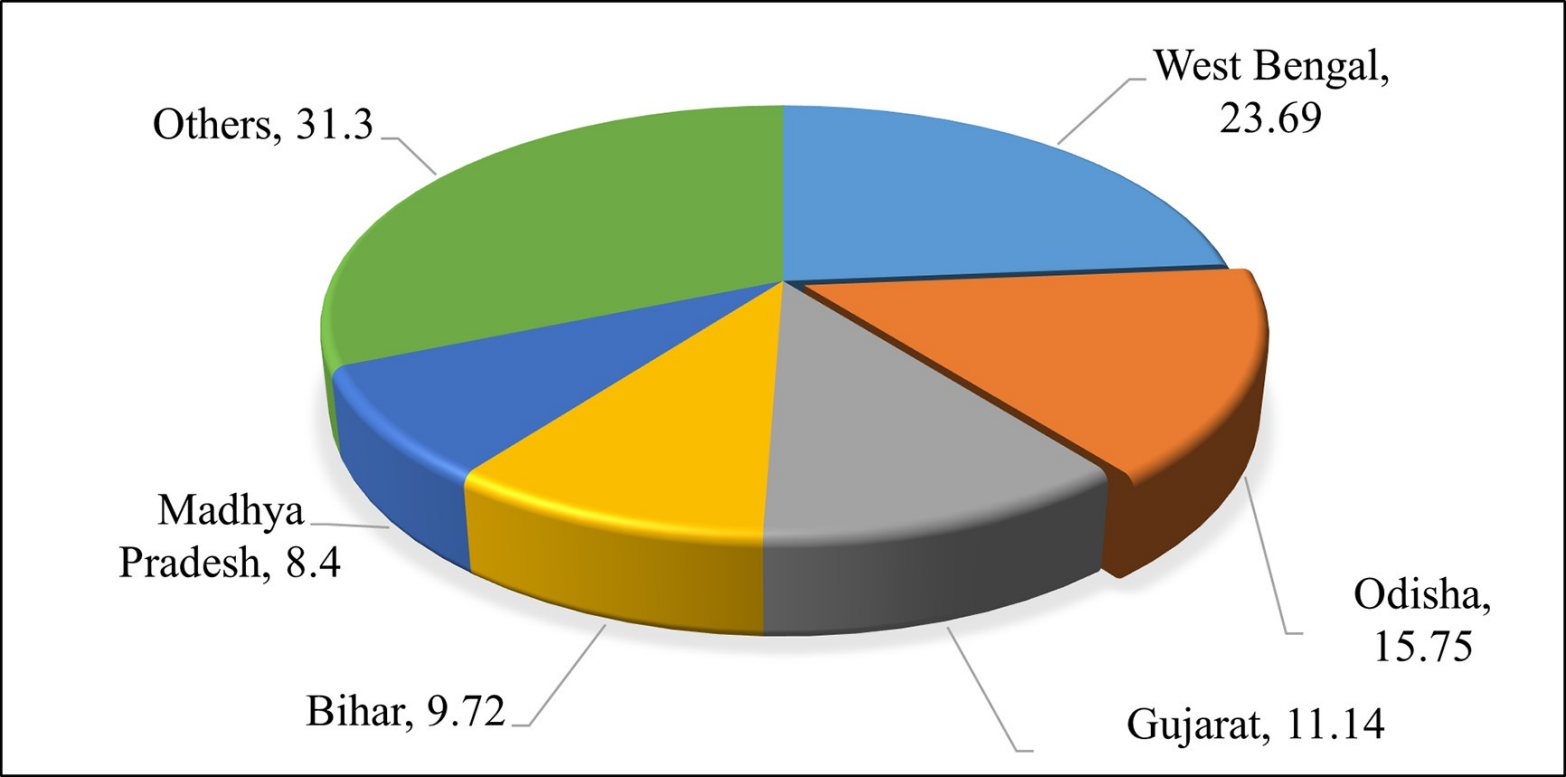
Percentage share of production of brinjal.

Agriculture plays a vital role in the Indian economy. Over 70 per cent of the rural households depend on agriculture. Agriculture is an important sector of Indian economy as it contributes about 20% to the total GDP and provides employment to over 60% of the population. Indian agriculture has registered impressive growth over last few years [1]. The continuous supply of agricultural commodities. Horticulture sector encompasses a wide range of crops like fruits, vegetables, flowers, spices, plantation crops like coconut, beverages like tea and coffee and some medicinal and aromatic plants. Statistics provided by National Horticulture Board shows that India accounting for 57.31% of the total production of vegetables and 6.92% brinjal (Horticultural Statistics at a Glance 2018). Brinjal is one of the most common tropical vegetables grown in India. Brinjal is a very nutritive vegetable that provides 52.0 mg of chlorine, 47.0 mg of phosphorus, 44.0 mg of Sulphur, 6.4 mg of vitamin A, 18.0 mg of Calcium, 24 k cal of energy, 1.3 g of fiber, 0.9 mg of iron, 1.4 g of protein, 12.0 mg of vitamin C, and 18.0 mg of oxalic acid, nutrients also available from a 100g of brinjal [2]. Odisha ranks 4^th^ position as far as production of vegetable is concerned in National level. Brinjal is a native of India, and is cultivated across many states in large scale and consumed by almost all household. In 2017–18, as per the records of National Horticulture Database, the area under brinjal production was 1.17ha, with production of 20.13 lakh tonnes and productivity of 17.07 mt/ha. As far as brinjal production is concerned, with 15.75% share of production in the national level, Odisha ranks 2^nd^ after West Bengal (Fig 1). The marketing decisions could be enriched with correct price forecasts for maximizing the returns and reducing the risk.

The price and arrival data information improve the bargaining position of the farmers and improves competition between traders. When the information on price is available, the farmer remains in a better position to switch between the alternative nearby markets to dispose the produce and getting good prices for their products. The farmers can use the information to make choices around the timing of marketing. Consequently, erratic price variations should be reduced as arbitrage over time and space becomes easier and more widespread [3]. A significant characteristic of vegetable price series is the seasonality, which is the biggest obstacle for obtaining accurate forecasts of vegetable prices. Given the complexity of the price series, many models have been specified for capturing the behavior of vegetable prices, but researchers have not reached a consensus on the best model for vegetable prices [4].

Within time series framework, many linear and nonlinear approaches, such as Autoregressive integrated moving average (ARIMA) model, Seasonal ARIMA (SARIMA), Generalized autoregressive conditional heteroscedastic (GARCH) model have been developed. In past, many studies have been conducted with the objective of predicting agricultural commodity prices [5–9]. It is reported that SARIMA model outperforms other price forecasting models for forecasting onion prices in Mumbai markets [5]. Application of SARIMA model for forecasting meat exports from India can be found in [6]. Price volatility in agricultural commodity in Inida has been extensively studied in [7]. Forecasting of Retail Price of Arhar Dal in Karnal market of Haryana has been carried out using stochastic models [8]. Different statistical models for forecasting volatility in onion price in selected markets of Delhi have been studied in [9].

In recent times, algorithms of Machine Learning (ML) which have developed within data science paradigm [10] has been dominated. It has been applied to forecasting financial and economic time series [11, 12]. Results of numerous empirical studies have shown that ML approaches outperform time series models in forecasting different financial assets [13]. A comparative analysis of statistical models and machine learning techniques can be found in [14]. Among the ML techniques, Artificial Neural Network (ANN), Generalized Neural Network (GRNN), Support Vector Regression (SVR), Random Forest (RF) and Gradient Boosting Machine (GBM) etc. are widely used. All these techniques are data-driven nonparametric techniques which learn the stochastic dependency in the data. It is reported that ANNs outperform the classical statistical methods such as linear regression and Box- Jenkins approaches [15]. GRNN is considered to be a promising alternative to the linear and nonlinear time series models [16]. Different intelligent models; namely, ANN, SVR, and extreme learning machine (ELM) have been applied for forecasting of bean and pig grain products [17]. With high seasonality [18, 19], reported that, machine learning and deep learning-based algorithms are the efficient approaches for solving time series prediction problems. The superiority of neural network over statistical methods is established in predicting agricultural prices [20]. Strategies in forecasting time series using GRNN by taking the advantage of their inherent properties to generate fast, highly accurate forecasts are nicely described in [21]. SVR was applied in predicting hog prices [22]. SVR has been used in financial time series forecast and exchange rate forecasting [23, 24]. GBM algorithm was applied to deal with the time series prediction tasks for coastal bridge engineering [25]. A random forest based regression model was developed in [26] to predict daily evapotranspiration from *in-situ* meteorological data and fluxes, satellite leaf area index (LAI), and land surface temperature data and found that the LAI is the most important feature. An application of Random Forests regression for Crop Yield Predictions may be found in [27]. For some theoretical developments of modeling time series data using random forest, one may refer to [28].

But in most of the studies either weekly or monthly price data has been considered. In averaging the data to compute weekly or monthly series, the actual variability present in the data is not truly represented. In ML algorithms, depending on the purpose of the analysis, logic of modeling on the basis of available data is build up. This avoids the complex and lengthy pre-model stage of statistical testing of various hypotheses about studied process. The main objective of present paper is to compare the predictive accuracies of the efficient ML algorithms: GRNN, SVR, RF and GBM for forecasting wholesale price of Brinjal in major markets of Odisha, India. Unlike other previous studies, here daily data has been considered and variation in prices of brinjal in almost all the major vegetables markets of Odisha, India have been taken into consideration. The hypothesis addressed in the present investigation is to determine best forecast model in terms of prediction accuracy. The performance comparison of different models has been carried out form many angles including, Circular plot, Radar plot, the model comparison set, Diebold Mariano test and other statistical measures e.g. RMSE, MAPE etc.

## Materials and methods

### Autoregressive integrated moving average (ARMA) model

Amongst the linear time series models, the Box-Jenkin’s Autoregressive Moving Average (ARMA) model has been widely used in the empirical literature. The ARIMA model denoted as ARIMA(p, d, q) is given by

$$\varphi (B){\left(1-B\right)}^{d}y_{t}=\theta (B)\varepsilon_{t}$$

Where ∅(*B*), *θ*(*B*) are the Autoregressive and Moving average polynomial as defined by:

$$\emptyset (B)=1-\emptyset_{1}B-\emptyset_{2}B^{2}-\cdots \emptyset_{P}B^{P}$$

and

$$\theta (B)=1-\theta_{1}B-\theta_{2}B^{2}-\cdots -\theta_{P}B^{P}$$

In the above, B is the backshift operator, i.e., *By*_*t*_ = *y*_*t*−1_, p and q are the order of autoregressive and moving average respectively. *ε*_*t*_ is a white noise process.

### Support vector regression (SVR)

For a given data set $D={\left\{(x_{i},y_{i})\right\}}_{i=1}^{N}$, where *x*_*i*_∈*R*^*n*^ input vector is, *y*_*i*_∈*R* is scalar output and N corresponds to size of data set, general form of Nonlinear SVR estimating function is:

$$f(x)=w^{T}\varphi (x)+b$$

Where $\varphi (.):\;R^{n}\to R^{n_{h}}$ is a nonlinear mapping function from original input space into a higher dimensional feature space, which can be infinitely dimensional, $w\in R^{n_{h}}$ is weight vector, *b* is bias term and superscript T indicates transpose. The coefficients *w* and *b* are estimated from data by minimizing the following regularized risk function:

$$R(\theta )=\frac{1}{2}{\left|\left|w\right|\right|}^{2}+C\left[\frac{1}{N}\sum_{i=1}^{N}L_{\varepsilon }(y_{i},f(x_{i}))\right].$$

In above equation, first term $\frac{1}{2}{\left||w|\right|}^{2}$ is called ‘regularised term’, which measures flatness of the function. Second term $\frac{1}{N}\sum_{i=1}^{N}L_{\varepsilon }(y_{i},f(x_{i}))$ called ‘empirical error’ is estimated by Vapnik *ε*-insensitive loss function. Both *C* and *ε* are user-determined hyper-parameter. Here, the Vapnik Loss function is given by:

$$L_{\varepsilon }(y_{i},f(x_{i}))=\left\{ \begin{array}{c} |y_{i}-f(x_{i})|-\varepsilon \;|y_{i}-f(x_{i})|\geq \varepsilon \\ \;0\;|y_{i}-f(x_{i})|<\varepsilon \end{array}\right.$$

**Fig 2:**
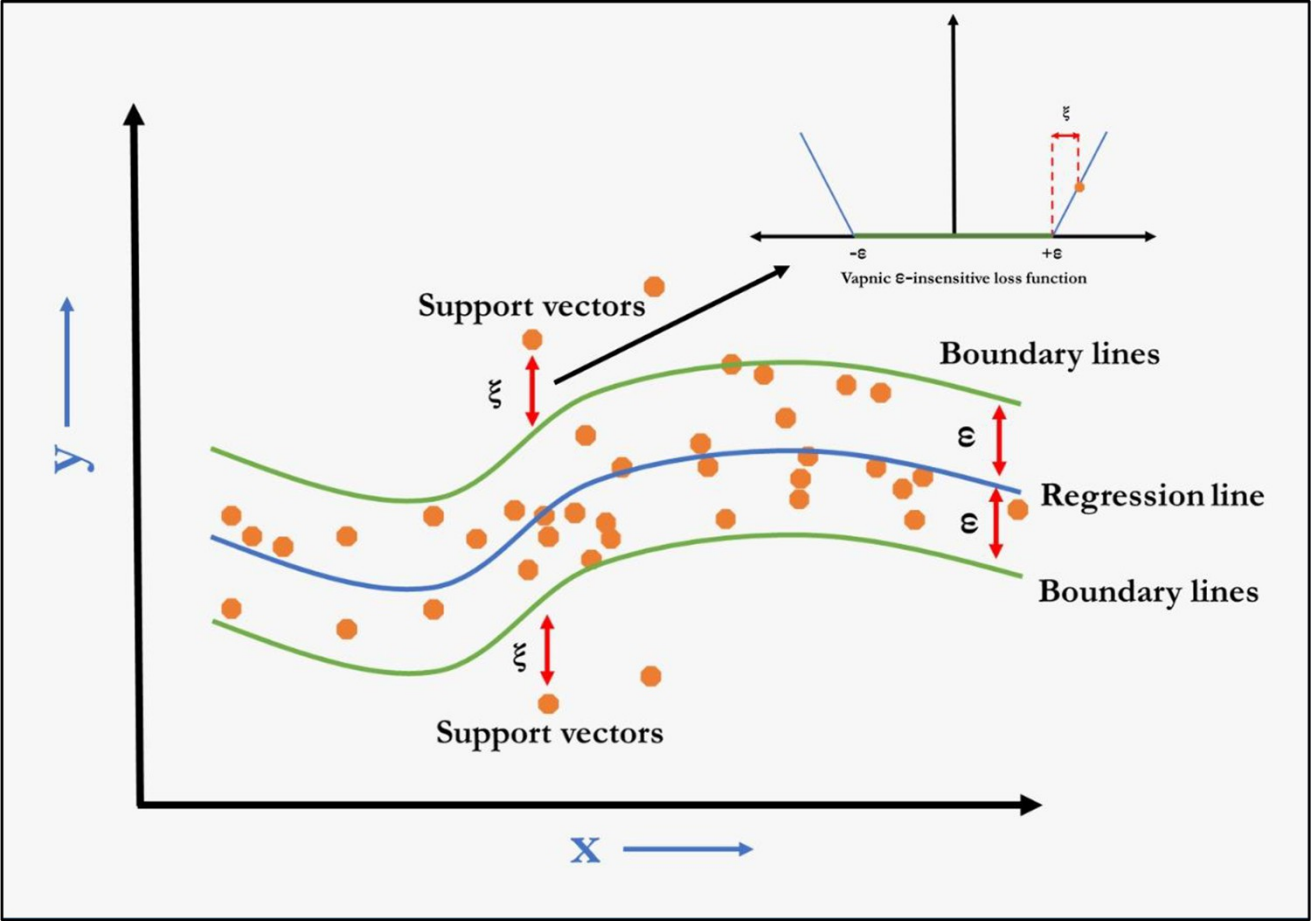
The algorithm of SVR.

Where *y*_*i*_ denotes actual value and i*f*(*x*_*i*_) is the estimated value at *i*th period. The algorithm of SVR is depicted in Fig 2. Few applications of nonlinear SVR in forecasting time series may be found in [29, 30].

### Random forest (RF)

**Fig 3:**
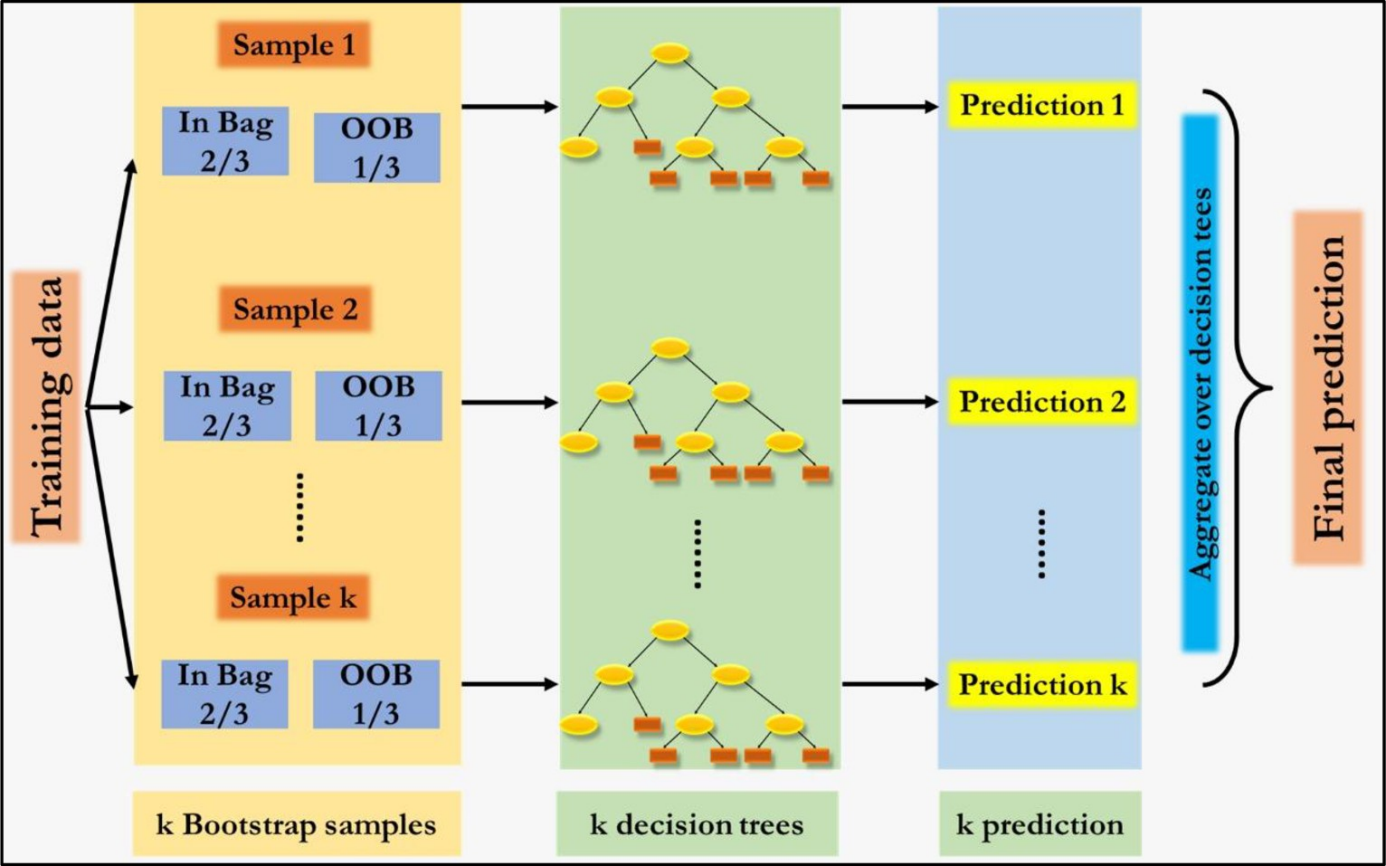
The schematic representation of RF.

Random forest is based on bagging technique (bootstrap aggregation) over decision trees [31]. Bagging reduces the variance of the base algorithms when they are weakly correlated. It is a flexible, easy to use supervised machine learning algorithm. It is also one of the most used algorithms, because of its simplicity and diversity. The benefits of bagging in forecasting time seirs have been listed in [32]. Random forest builds multiple decision trees and merges them together to get a more accurate and stable prediction. In RF the correlation between trees is reduced by randomization in two directions. Firstly, each tree is trained on a bootstrapped subset. Secondly, the feature by which splitting is performed in each node is not selected from all possible features, but only from their random subset of size m. The RF algorithm generates each of the N trees independently, which makes it very easy to parallelize. For each tree, it constructs a full binary tree of maximum depth. Thereby efficiency of RF performance is achieved. The schematic representation of RF is given in Fig 3. Here, OOB stands for Out-of-Bag sample.

### Generalized regression neural network (GRNN)

**Fig 4:**
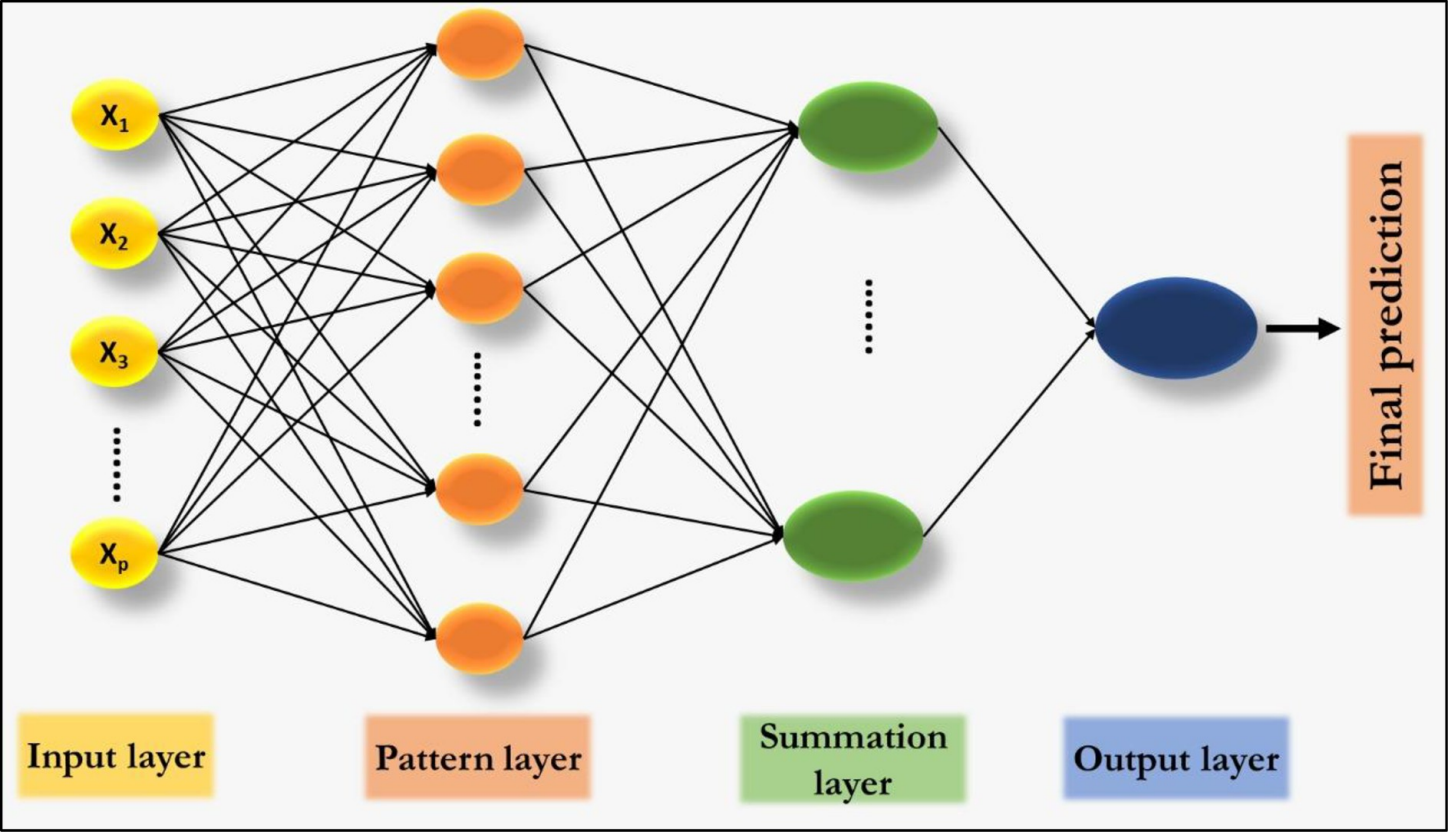
The schematic representation of GRNN.

Generalized regression neural network is related to the radial basis neural networks, which are found on kernel regression. It can be treated as a normalized radial basis neural networks in which there is a hidden neuron centered at every training case. These radial basis function units are generally probability density function such as the Gaussian [33]. GRNN approximates any arbitrary function between input and target vectors; fast training and convergence to the optimal regression surface as the training data becomes very large [34]. This makes GRNN a very advantageous tool to perform predictions. The GRNN architecture as depicted in Fig 4 has four layers: an input layer, a hidden layer, a summation layer, and an output layer. The hidden layer has radial basis neurons with training examples as centers. The output of hidden layer neuron is linked to the nearness of the input vector to the center, scaled by the smoothing parameter [21].

### Gradient Boosting Machine (GBM)

**Fig 5:**
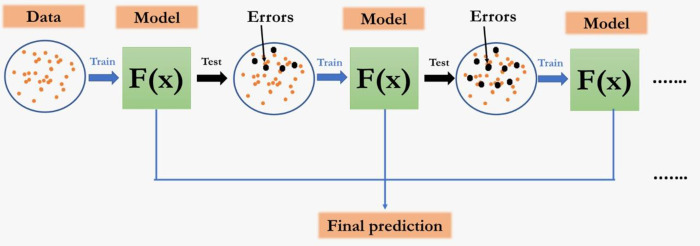
The sequential procedure in GBM.

Gradient Boosting Machine method (GBM) proposed by [35, 36] is a boosting algorithm used when dealing with plenty of data to make a prediction with high prediction power. Boosting is actually an ensemble of learning algorithms which combines the prediction of several base estimators in order to improve robustness over a single estimator. It follows the procedure of sequentially building a composition of machine learning algorithms, when each of them seeks to compensate for the shortcomings of the composition of all previous algorithms. Compared to bagging, boosting does not use simple voting but a weighted one. It combines multiple weak or average predictors to a build strong predictor. In GBM, the nodes in every decision tree take a different subset of features for selecting the best split. This means that the individual trees aren’t all the same and hence they are able to capture different signals from the data. Additionally, each new tree takes into account the errors or mistakes made by the previous trees. So, every successive decision tree is built on the errors of the previous trees. This is how the trees in a gradient boosting machine algorithm are built sequentially. The procedure is depicted in Fig 5.

### Validation of forecasts

The dataset for each market was divided in two parts before analysis with 90% of the observations for estimation (model development) and remaining 10% for validation purpose. Comparative assessment of prediction performance of different models namely ARIMA, RF, GRNN, GBM and SVR models was carried out in terms of mean error (ME), Mean absolute error (MAE), root mean square error (RMSE) and Mean absolute percentage error (MAPE) based on the following formulae:

$$ME=1/h\sum_{i=1}^{h}\left\{y_{t+i}-{\hat{y}}_{t+i}\right\}$$

$$MAE=1/h\sum_{i=1}^{h}\left|y_{t+i}-{\hat{y}}_{t+i}\right|$$

$$RMSE=\sqrt{1/h{\sum_{i=1}^{h}\left\{y_{t+i}-{\hat{y}}_{t+i}\right\}}^{2}}$$

$$MAPE\;(\%)=1/h\sum_{i=1}^{h}|\frac{y_{t+i}-{\hat{y}}_{t+i}}{y_{t+i}}|\times 100$$

where h denotes the number of observations for validation, y_i_ is the observed value and ${\hat{y}}_{i}$ is the predicted one. Diebold Mariano test [37] was also conducted for different pairs of models to test for differences in predictive accuracy between any two competing models. Beside these, MCS [38, 39] has been used to find the superior set of models for prediction. To strengthen our claim on the superiority of the model, circular plot and radar plots [40] have also been utilized.

## Result and discussion

### Dataset

Daily wholesale price data of Brinjal for the period 1^st^ Jnauary, 2015 to 31^st^ May, 2021 have been collected for seventeen different markets of Odisha, India from AGMARKNET (https://agmarknet.gov.in/). The portal is run by the Directorate of Marketing & Inspection, Government of India. The agriculture produce markets enters the data using the customized application software “Agmark”. Before analysis, the missing observations were imputed using suitable statistical techniques.

### Data description

**Fig 6:**
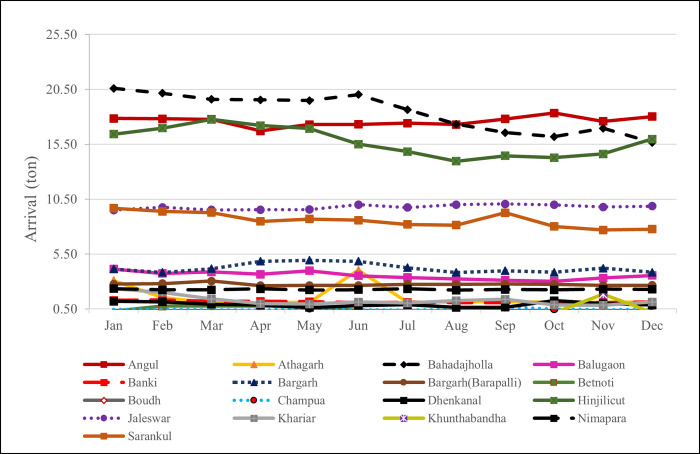
Average monthly arrival of brinjal in important markets of Odisha.

**Fig 7:**
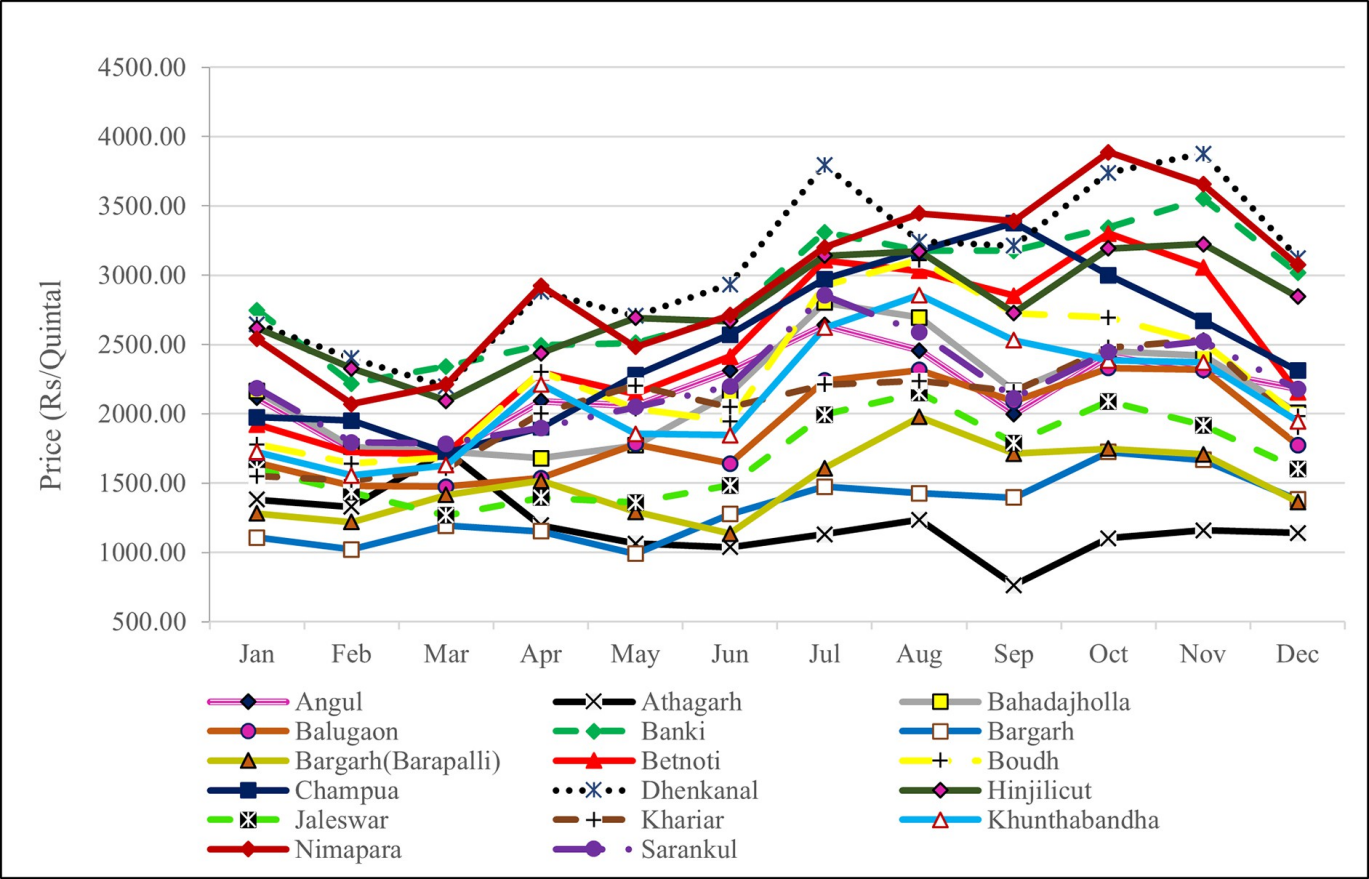
Average monthly price of brinjal in important markets in Odisha.

The overall summary statistics of the price data are reported in Table 1. A perusal of Table 1 indicates that average price remains high in Dhenkanal market whereas lower average price is observed in Bargarh. Angul experienced maximum price (Rs 7500/quintal) as well as minimum price (Rs 250/quintal during the study period. The kurtosis is higher in almost all the markets leading to platykurtic nature of distribution. The variability in price series as observed by coefficient of variation (CV) ranges between minimum 25.25% in Hinjilicut to maximum of 95.41% in Athagarhmarket. The Jarque-Bera of normality indicated that all the market prices follow non-normal distribution. The price and arrival of Brinjal in various markets in the state of Odisha (Figs 6 and 7; Tables 2 and 3) gives an indication of demand and supply. There is potentiality for growing all types of tropical, sub-tropical and temperate vegetable in the region. The Individual market commodity arrival also depends on the infrastructure and handling capacity of any commodity in the specific market. It is also an indication of the production of commodity in that particular region. These indications need to be taken into consideration by the farmers in production capacity useful resources in the market. It is observed that the Bahadajholla (220.76 ton) receives largest volume of brinjal among the seventeen markets and is followed by Angul (211.24 ton), Hinjilicut (188.95 ton), Jaleswar 117.34 ton) and Sarankul (103.17 ton). Apart from these markets we are having some other market recorded very less quantity of brinjal arrival data i.e. Khunthabandha and Boudh market arrival data (3.70 and 3.92 ton) respectively. Thus, it can be concluded that the farmers of Odisha could take the commodity in regional markets like Bahadajholla, Angul,Hinjilicut and Jaleswar. The season wise analysis of arrival of brinjal in major markets of north eastern India defines that in the Bahadajholla market the arrivals are low during the months of October to December and during September to November. In Angul market the supply of brinjal during the April is lowest.

**Table 1:** Summary statistics.

Market	Mean	Median	Maximum	Minimum	Std. Dev.	Skewness	Kurtosis	CV	Jarque-Bera	Probability
Angul	2156.274	2500	7500	250	632.86	0.14	4.74	29.35	302.92	<0.001
Athagarh	906.5514	380	4400	250	866.21	1.47	4.48	95.41	1062.61	<0.001
Bahadajholla	2130.662	2000	5000	300	807.10	0.83	4.01	37.88	368.70	<0.001
Balugaon	1869.074	1700	4200	520	698.68	0.96	3.46	37.38	378.27	<0.001
Banki	2854.802	2800	6800	750	843.41	0.63	4.34	29.54	332.10	<0.001
Bargarh	1303.713	1300	4300	400	543.39	1.12	5.71	41.68	1206.72	<0.001
Bargarh_barapalli	1488.412	1200	4800	400	692.75	1.18	4.21	46.54	685.77	<0.001
Betnoti	2446.863	2200	6000	500	1018.64	0.63	3.10	41.63	154.34	<0.001
Boudh	2257.337	2000	5000	700	814.72	0.45	2.70	36.09	88.32	<0.001
Champua	2459.605	1850	5950	900	1179.86	1.49	4.28	47.97	1024.88	<0.001
Dhenkanal	3034.528	3000	6500	500	1075.35	0.22	2.77	35.44	23.82	<0.001
Hinjilicut	2743.227	2800	5000	800	692.72	0.02	2.85	25.25	2.28	<0.001
Jaleswar	1658.188	1500	4300	500	683.40	1.20	4.59	41.21	812.47	<0.001
Khariar	2023.624	2000	4100	600	674.61	0.73	3.36	33.34	218.07	<0.001
Khunthabandha	2109.134	2000	4000	500	736.53	0.49	2.99	34.92	92.87	<0.001
Nimapara	2936.769	2900	6000	800	1015.08	0.32	2.35	34.56	80.47	<0.001
Sarankul	2202.582	2200	5200	600	743.75	0.88	4.58	33.77	545.66	<0.001

**Table 2:** Average monthly arrival of brinjal in important markets in Odisha.

Market	January	February	March	April	May	June	July	August	September	October	November	December
Angul	17.85	17.81	17.77	16.71	17.29	17.32	17.42	17.29	17.81	18.34	17.59	18.03
Athagarh	3.09	1.53	1.05	1.07	1.11	4.03	1.12	1.13	1.17	1.10	1.13	1.15
Bahadajholla	20.60	20.15	19.59	19.55	19.49	20.02	18.65	17.33	16.56	16.19	16.96	15.66
Balugaon	4.14	3.73	3.88	3.66	3.99	3.53	3.36	3.23	3.13	3.03	3.32	3.56
Banki	1.28	1.32	1.21	1.20	1.16	1.08	1.11	1.07	1.07	1.09	1.09	1.13
Bargarh	4.13	3.83	4.17	4.84	4.94	4.84	4.26	3.82	3.98	3.84	4.22	3.84
Bargarh(Barapalli)	2.77	2.82	3.05	2.62	2.64	2.64	2.75	2.73	2.77	2.75	2.65	2.66
Betnoti	0.31	0.76	0.78	0.78	0.68	0.36	0.18	0.19	0.29	0.23	0.28	0.32
Boudh	0.34	0.34	0.33	0.29	0.33	0.33	0.31	0.31	0.33	0.33	0.34	0.34
Champua	0.47	0.45	0.44	0.41	0.56	0.44	0.42	0.46	0.68	0.51	0.43	0.46
Dhenkanal	1.20	1.16	0.93	0.86	0.63	0.82	0.88	0.64	0.66	1.26	0.99	0.85
Hinjilicut	16.42	16.97	17.75	17.21	16.94	15.51	14.84	13.96	14.45	14.29	14.63	15.98
Jaleswar	9.51	9.75	9.53	9.54	9.55	10.00	9.74	10.00	10.06	9.99	9.78	9.87
Khariar	2.35	2.01	1.41	0.98	0.95	1.14	1.04	1.27	1.39	0.88	0.84	1.14
Khunthabandha	0.19	0.19	0.17	0.16	0.16	0.18	0.16	0.14	0.15	0.17	1.84	0.18
Nimapara	2.35	2.25	2.25	2.34	2.23	2.26	2.34	2.22	2.27	2.24	2.30	2.29
Sarankul	9.68	9.38	9.28	8.48	8.70	8.58	8.20	8.13	9.26	8.01	7.69	7.77

**Table 3:** Average monthly price of brinjal in important markets in Odisha.

Market	January	February	March	April	May	June	July	August	September	October	November	December	Average Price
Angul	2114.75	1731.57	1699.54	2091.90	2057.83	2310.28	2639.25	2455.91	1997.78	2412.90	2310.00	2178.49	2166.68
Athagarh	1380.32	1328.08	1734.24	1195.33	1064.93	1037.39	1129.25	1236.67	762.87	1102.47	1159.61	1140.91	1189.34
Bahadajholla	2162.21	1763.13	1725.58	1680.24	1771.89	2145.83	2801.72	2694.09	2165.11	2453.49	2416.94	2006.99	2148.94
Balugaon	1647.24	1480.56	1474.88	1535.81	1780.65	1640.56	2238.44	2316.40	2089.44	2330.91	2318.89	1772.96	1885.56
Banki	2746.31	2218.18	2341.94	2493.81	2510.14	2693.33	3309.68	3177.96	3175.56	3343.01	3552.22	3016.13	2881.52
Bargarh	1105.30	1020.20	1191.24	1152.14	988.94	1276.67	1474.73	1426.88	1395.83	1724.73	1666.94	1380.65	1317.02
Bargarh(Barapalli)	1280.88	1217.93	1413.82	1515.71	1292.17	1136.11	1608.33	1977.42	1711.67	1748.66	1708.33	1362.90	1497.83
Betnoti	1922.58	1716.16	1714.29	2300.00	2144.70	2414.44	3108.06	3030.11	2853.33	3299.46	3056.39	2152.42	2476.00
Boudh	1780.37	1641.92	1686.64	2303.33	2035.94	1947.22	2923.66	3110.75	2725.00	2695.70	2513.89	1984.95	2279.11
Champua	1973.53	1949.49	1716.22	1899.86	2278.39	2571.94	2970.97	3177.15	3376.53	2998.66	2670.97	2311.56	2491.27
Dhenkanal	2646.54	2402.02	2196.77	2881.43	2705.99	2934.44	3795.70	3244.09	3211.11	3739.25	3877.22	3122.58	3063.10
Hinjilicut	2619.12	2327.65	2093.09	2436.19	2692.17	2668.33	3140.32	3170.43	2728.33	3192.47	3223.78	2845.16	2761.42
Jaleswar	1599.91	1434.29	1267.70	1394.52	1359.77	1484.11	1992.96	2151.83	1787.58	2090.16	1917.33	1601.51	1673.47
Khariar	1549.77	1519.19	1605.53	2001.90	2202.76	2048.61	2210.75	2237.63	2161.67	2478.49	2531.67	1903.49	2037.62
Khunthabandha	1723.50	1554.04	1628.57	2213.33	1855.30	1844.44	2619.89	2857.53	2531.11	2384.41	2369.44	1946.51	2127.34
Nimapara	2539.63	2070.71	2211.52	2923.33	2483.41	2711.94	3200.00	3445.70	3390.56	3887.63	3656.11	3073.12	2966.14
Sarankul	2183.41	1793.18	1783.18	1898.81	2048.62	2198.89	2854.30	2587.37	2111.67	2447.04	2522.22	2176.34	2217.09

### Fitting of models

The stochastic model i.e. ARIMA and machine learning techniques e.g. RF, SVR, GRNN and GBM as described in methodology sections have been fitted for the data under consideration. Dependency of current price is taken upto 5 lags for all the markets. The lagged prices were considered as the exogenous variables in machine learning techniques. The preliminary order of ARIMA model was selected based on pattern of Autocorrelation function (ACF) and Partial autocorrelation function (PACF). The best fitted ARIMA model was chosen based on information criterion like Akaike Information Criterion (AIC), Schwartz Bayesian Criterion (SBC) and Hannan–Quinn information criterion (HQIC). The training of machine learning techniques has been carried out by optimizing the parameters and hyper parameters.

## Discussions

**Fig 8:**
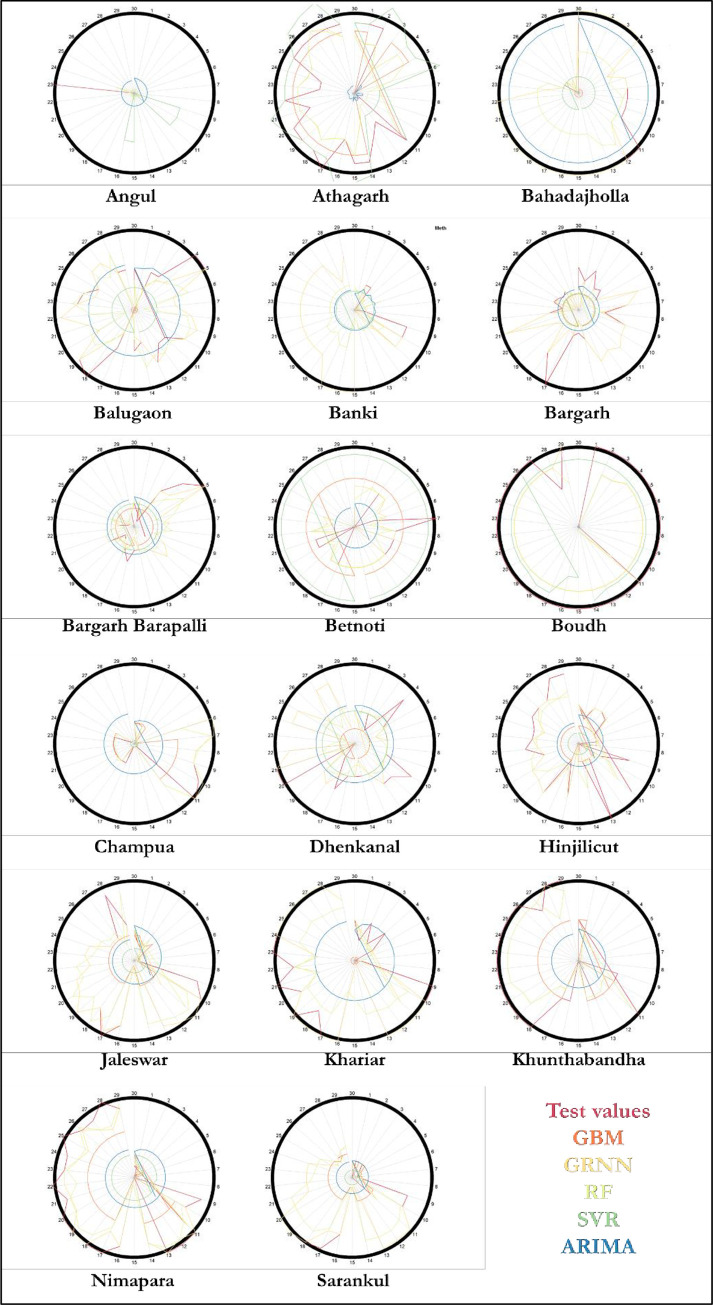
Circular plot.

**Fig 9:**
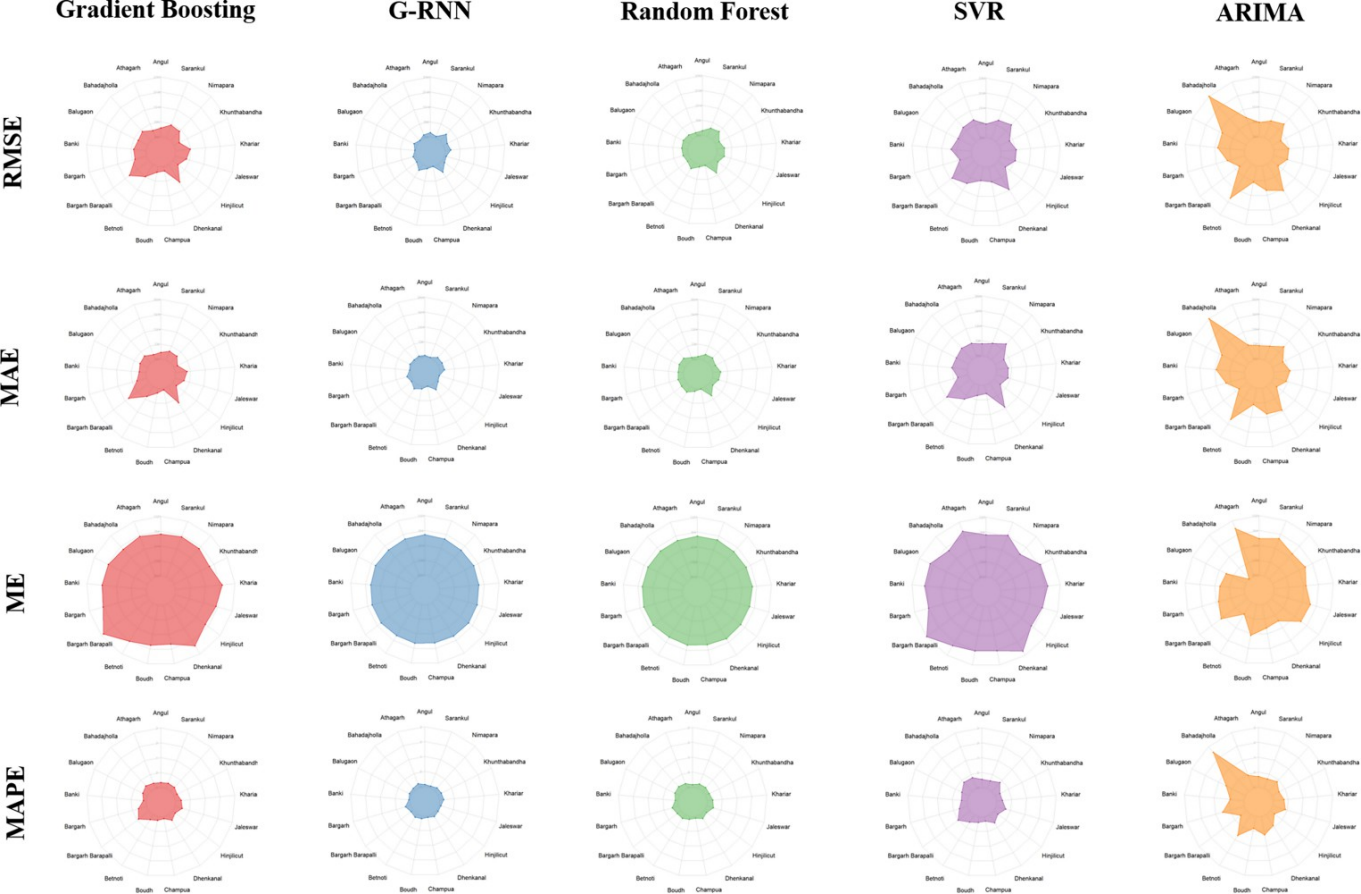
Radar plot.

The result of prediction performance measured by four statistics namely ME, MAE, RMSE and MAPE computed by the formulae described in the above section are reported in Table 4. A perusal of Table 4 indicates that, for all the markets, machine learning techniques perform better than that of usual ARIMA model. Among the four machine learning techniques used, in almost all the markets, GRNN performs better based on the above mentioned statistical measures except for Khunthabandha market where GBM performs better though the difference in gain in accuracy over GRNN and RF is not significant. The superiority set of models as found out by MCS is reported in Table 5. Among the 17 markets, in 14 markets it is observed that GRNN model is the superior model than that of other models; where as in remaining 4 markets namely Athagarh, Betnoti, Boudh and Khunthabandha, it is found that GRNN and RF performs at par and are superior models. To this end Diebold Mariano test was also conducted for different pairs of models to test for differences in predictive accuracy between any two competing models and it revealed that the predictive accuracy of GRNN is better than that of other technique in all the markets under consideration (Table 6). In four markets namely Athagarh, Betnoti, Boudh and Khunthabandha, the predictive accuracy of GRNN and RF don’t differ significantly. To visualize the prediction performance of different models, the Circular plots and Radar plots have been obtained and depicted in Figs 8 and 9 respectively. For circular plot, 30 steps ahead forecast (one month) has been depicted and it is observed that GRNN prediction is closest to the test value in majority of the markets while in few markets the prediction by GRNN and RF overlaps. The radar plots obtained based on all the four statistical measures i.e. ME, MAE, RMSE and MAPE represent the superiority of GRNN over other competing models.

**Table 4:** Prediction performance of different models.

	Angul				Atdagarh				Bahadajholla			
	ME	RMSE	MAE	MAPE	ME	RMSE	MAE	MAPE	ME	RMSE	MAE	MAPE
ARIMA	-172.02	858.05	656.71	35.34	767.13	1169.19	767.61	45.67	-2550.84	2762.54	2603.78	175.58
G-RNN	79.60	436.65	161.10	6.14	38.00	397.01	171.94	12.78	-6.14	285.59	144.52	8.41
Gradient Boosting	159.90	636.09	380.00	16.14	272.24	585.81	340.80	18.57	119.47	783.75	499.71	24.56
Random Forest	81.90	454.49	206.48	8.24	38.05	420.61	214.71	15.80	48.57	490.97	365.02	18.99
SVR	166.78	831.16	598.14	27.22	688.61	1107.92	703.74	38.92	78.26	1064.64	745.50	40.40
	Balugaon				Banki				Bargarh			
ARIMA	-1121.08	1368.76	1223.49	71.39	-952.19	1388.02	1289.36	44.96	-769.43	1029.30	920.42	75.13
G-RNN	65.28	399.79	175.36	8.06	8.72	287.63	123.07	3.68	43.25	388.76	256.62	17.86
Gradient Boosting	299.14	695.68	456.54	17.72	315.46	781.13	388.90	8.70	385.44	764.84	520.65	27.88
Random Forest	61.18	536.79	357.97	16.30	79.13	514.98	310.28	8.06	103.76	482.74	345.72	21.08
SVR	547.45	956.14	674.02	25.33	487.24	1115.48	749.43	18.85	369.31	778.31	530.70	28.98
	Bargarh Barapalli				Betnoti				Boudh			
ARIMA	-412.03	673.78	541.12	25.03	-1666.12	1936.14	1742.61	82.04	-505.24	942.73	802.21	40.04
G-RNN	38.56	355.36	180.88	7.76	-32.41	540.85	323.21	12.70	12.61	357.81	217.36	9.44
Gradient Boosting	1167.57	1283.74	1170.35	44.01	367.04	861.04	585.85	18.60	95.33	514.03	291.06	11.67
Random Forest	75.90	424.83	339.18	13.98	-46.56	551.06	372.50	15.04	15.98	358.21	219.10	9.46
SVR	1322.58	1426.22	1322.71	50.21	603.47	1164.79	881.67	28.51	400.40	836.81	544.77	18.85
	Champua				Dhenkanal				Hinjilicut			
ARIMA	-991.75	1295.20	1205.51	62.21	-1155.11	1564.54	1286.39	43.09	-122.76	637.43	396.70	16.55
G-RNN	-15.51	264.64	103.39	4.72	18.29	637.13	371.78	10.74	51.57	386.11	173.13	6.16
Gradient Boosting	27.78	451.75	157.42	6.12	708.82	1127.47	889.98	20.82	116.06	533.87	292.69	10.19
Random Forest	-8.02	291.23	123.65	5.33	73.63	754.86	553.14	15.70	22.50	452.73	258.90	9.04
SVR	388.93	898.74	462.08	14.68	1022.27	1465.59	1280.02	31.12	168.88	650.44	442.38	16.63
	Jaleswar				Khariar				Khunthabandha			
ARIMA	-58.10	867.71	661.20	43.57	-458.78	882.60	780.23	36.23	-176.22	649.31	588.68	26.62
G-RNN	22.53	330.44	104.92	5.84	2.46	501.21	304.48	13.84	30.95	428.15	285.65	11.97
Gradient Boosting	223.20	754.84	489.42	24.87	499.76	871.42	581.67	18.96	23.91	497.17	258.70	10.73
Random Forest	12.40	594.92	379.62	22.32	80.61	573.89	435.55	17.92	31.56	433.84	291.31	12.12
SVR	288.52	917.79	613.66	33.09	511.77	910.60	585.91	18.71	431.84	739.57	507.10	17.26
	Nimapara				Sarankul							
ARIMA	-336.33	1187.24	1013.04	41.96	67.18	1010.12	720.31	32.70				
G-RNN	-14.94	625.81	277.95	10.49	42.30	324.36	110.07	4.51				
Gradient Boosting	177.15	792.56	484.90	17.52	237.65	838.61	493.29	18.49				
Random Forest	4.91	692.22	386.49	14.75	84.87	619.27	352.55	14.11				
SVR	-233.11	1211.55	985.58	39.64	415.93	1091.01	702.74	27.63				

**Table 5:** Model Confidence Set (MCS) test results.

Markets	Best model(s)	p-value	Markets	Best model(s)	p-value
Angul	GRNN	0.025	Champua	GRNN	0.040
Athagarh	GRNN, RF	0.171	Dhenkanal	GRNN	0.000
Bahadajholla	GRNN	0.000	Hinjilicut	GRNN	0.000
Balugaon	GRNN	0.000	Jaleswar	GRNN	0.000
Banki	GRNN	0.003	Khariar	GRNN	0.002
Bargarh	GRNN	0.061	Khunthabandha	GRNN, RF	0.180
Bargarh Barapalli	GRNN	0.000	Nimapara	GRNN	0.001
Betnoti	GRNN, RF	0.537	Sarankul	GRNN	0.001
Boudh	GRNN, RF	0.790			

**Table 6:** Diebold Mariano test result.

	¶GRNN vs RF		GRNN vs SVR		GRNN vs GBM		GRNN vs ARIMA		RF vs SVR		RF vs GBM		RF vs ARIMA		SVR vs GBM		SVR vs ARIMA		GBM vs ARIMA	
	*TS	*p	TS	p	TS	p	TS	p	TS	p	TS	p	TS	p	TS	p	TS	p	TS	p
Angul	-1.87	0.03	-11.05	0.00	-6.72	0.00	-11.34	0.00	-11.01	0.00	-6.71	0.00	-11.35	0.00	12.21	1.00	-1.02	0.15	-8.21	0.00
Athagarh	-1.16	0.12	-8.10	0.00	-3.54	0.00	-8.56	0.00	-8.10	0.00	-3.51	0.00	-8.56	0.00	9.71	1.00	-12.35	0.00	-10.13	0.00
Bahadajholla	-6.11	0.00	-6.83	0.00	-5.49	0.00	-28.24	0.00	-6.68	0.00	-4.92	0.00	-26.49	0.00	8.61	1.00	-17.59	0.00	-21.29	0.00
Balugaon	-4.62	0.00	-8.04	0.00	-6.69	0.00	-16.16	0.00	-6.66	0.00	-4.11	0.00	-14.16	0.00	8.53	1.00	-5.54	0.00	-10.42	0.00
Banki	-4.40	0.00	-7.00	0.00	-5.39	0.00	-21.41	0.00	-7.10	0.00	-5.00	0.00	-18.73	0.00	8.66	1.00	-3.56	0.00	-10.61	0.00
Bargarh	-3.12	0.00	-5.90	0.00	-5.75	0.00	-15.20	0.00	-6.43	0.00	-6.25	0.00	-13.30	0.00	8.34	1.00	-4.39	0.00	-4.63	0.00
Bargarh Barapalli	-4.02	0.00	-16.40	0.00	-14.37	0.00	-8.02	0.00	-16.50	0.00	-14.42	0.00	-7.29	0.00	34.49	1.00	12.91	1.00	10.72	1.00
Betnoti	-0.62	0.27	-7.06	0.00	-4.77	0.00	-17.34	0.00	-7.00	0.00	-4.70	0.00	-17.30	0.00	9.72	1.00	-7.99	0.00	-12.02	0.00
Boudh	-0.26	0.40	-7.36	0.00	-5.34	0.00	-14.18	0.00	-7.38	0.00	-5.37	0.00	-14.17	0.00	7.77	1.00	-2.09	0.02	-12.30	0.00
Champua	-1.87	0.03	-4.78	0.00	-1.74	0.04	-20.27	0.00	-4.83	0.00	-1.81	0.04	-20.87	0.00	5.00	1.00	-5.95	0.00	-18.53	0.00
Dhenkanal	-5.04	0.00	-10.28	0.00	-7.80	0.00	-9.78	0.00	-9.71	0.00	-6.99	0.00	-9.10	0.00	12.19	1.00	-0.98	0.16	-4.42	0.00
Hinjilicut	-4.39	0.00	-6.28	0.00	-4.62	0.00	-5.43	0.00	-5.20	0.00	-3.08	0.00	-4.29	0.00	5.38	1.00	0.67	0.75	-3.01	0.00
Jaleswar	-4.02	0.00	-6.43	0.00	-5.64	0.00	-7.67	0.00	-4.31	0.00	-2.85	0.00	-4.55	0.00	6.12	1.00	2.15	0.98	-5.35	0.00
Khariar	-3.29	0.00	-6.25	0.00	-6.04	0.00	-11.79	0.00	-5.90	0.00	-5.61	0.00	-10.37	0.00	6.48	1.00	0.52	0.70	-0.22	0.41
Khunthabandha	-1.34	0.09	-6.24	0.00	-4.37	0.00	-6.58	0.00	-6.24	0.00	-4.19	0.00	-6.52	0.00	5.80	1.00	2.48	0.99	-5.30	0.00
Nimapara	-2.56	0.01	-7.18	0.00	-3.35	0.00	-7.63	0.00	-6.63	0.00	-2.54	0.01	-6.93	0.00	8.17	1.00	1.86	0.97	-8.47	0.00
Sarankul	-4.31	0.00	-6.96	0.00	-6.18	0.00	-7.49	0.00	-5.17	0.00	-3.40	0.00	-5.02	0.00	7.05	1.00	3.60	1.00	-6.59	0.00

¶ the alternative hypothesis is: The predictive accuracy of first model is greater than the second one

*TS*p indicates test statistic and p value respectively

## Conclusions

The market arrival and price data of seventeen major markets in Odisha, India was analysed. The study of price of brinjal prevailing in major eastern Indian markets describe that the highest price prevails in Dhenkanal market (3063.10 Rs/Quintal) (Table 3). The average annual price of brinjal prevailing in Nimapara and Banki is 7.4% and 4.4%, respectively higher than that in Hinjilicut. Thus, the Hinjilicut farmers may approach to Banki, Nimapara and Dhenkanal for better price realization. The price behaviour based on seasonal index revealed that the highest price of brinjal prevails in the month of July and followed by October and November in Dhenkanal market. The lowest price is observed in September and December in the Athagarh market. Thus, it is revealed that the Athagarh market despite receiving low volume of brinjal compared to Nimapara and Banki markets provides opportunity for exploiting better price prevailing there. Therefore, forecast of market price of the agricultural produce will help farmers to gain more profit by taking the products to the nearby market where better price realization prevails. Based on the historical price pattern in different markets, the forecasting models were developed and it was found that ARIMA model cannot capture the variation in prices over time and the accuracy of this model is also not up to the mark. The machine learning techniques namely RF, GRNN, GBM and SVR performed better than that of ARIMA model in all the markets. Among the machine learning techniques used in the present study, GRNN has performed better than that of others in majority of the markets. The study revealed that if the model is trained properly with sufficient observations, we can achieve the desired accuracy in prediction using GRNN and other ML techniques. However, price of a commodity may depend on several exogenous factors including weather variables which have not been considered in the present study. Moreover, carrying the agriculture produce in distant markets offers various difficulties in terms of low quantities and related marketing risks and uncertainty. The farmers need to be empowered to be able to aggregate the product so as to exploit the economies of scale and take benefit of current institutional changes in agricultural marketing. In future study, some nearby markets may be selected for investigating spatial dependency and incorporating that dependency in developing model to check for any significant gain in accuracy.

## References

[1] Arjun KM. Indian agriculture-status, importance and role in Indian economy. *International Journal of Agriculture and Food Science Technology*, 2013: 4(4): 343–346.

[2] Choudhary B, Gaur K. *The development and regulation of Bt brinjal in India (Eggplant/Aubergine)*. International Service for the Acquisition of Agri-biotech Applications. 2009.

[3] Fafchamps M, Minten B. Impact of SMS-based agricultural information on Indian farmers. *The World Bank Economic Review*, 2012; 26(3): 383–414.

[4] Xiong T, Li C, Bao Y. Seasonal forecasting of agricultural commodity price using a hybrid STL and ELM method: Evidence from the vegetable market in China. Neurocomputing, 2018; 275, 2831–2844.

[5] Sankaran S. Demand forecasting of fresh vegetable product by seasonal ARIMA model. International Journal of Operational Research 20, 2014; (3): 315–330.

[6] Paul RK, Panwar S, Sarkar SK, Kumar A, Singh KN, Farooqi S, et al. Modelling and forecasting of meat exports from India. Agricultural Economics Research Review 2013; 26 (2): 249–256.

[7] Paul RK, Bhardwaj SP, Singh DR, Kumar A, Arya P, Singh KN. Price Volatility in Food Commodities in India- An Empirical Investigation. *International Journal of Agricultural and Statistical Sciences*, 2015; 11(2): 395–401.

[8] Paul RK, Gurung B, Paul AK. Modelling and forecasting of the retail price of arhar dal in Karnal, Haryana., (1), Indian Journal of Agricultural Science. 2015; 85: 69–72.

[9] Paul RK, Rana S, Saxena R. Effectiveness of price forecasting techniques for capturing asymmetric volatility for onion in selected markets of Delhi. Indian Journal of Agricultural Sciences 2016; 86(3): 303–309.

[10] Flach P. Machine Learning: The Art and Science of Algorithms that Make Sense of Data. 2012: Cambridge University Press, Cambridge.

[11] Bontempi G, Taieb S, Borgne Y. Machine Learning Strategies for Time Series Forecasting. Business Intelligence. Lecture Notes in Business Information Processing 2013; 138: 62–77. doi: 10.1007/978-3-642-36318-4_3

[12] Derbentsev V, Matviychuk A, Soloviev VN. Forecasting of Cryptocurrency Prices Using Machine Learning. In: Pichl L., Eom C., Scalas E., Kaizoji T. (eds.) Advanced Studies of Financial Technologies and Cryptocurrency Markets, 2020; pp. 211–231. Springer, Singapore. doi: 10.1007/978-981-15-4498-9_12

[13] Kumar D, Rath SK. Predicting the Trends of Price for Ethereum Using Deep Learning Technique. In: Dash S., Lakshmi C., Das S., Panigrahi B. (eds.) Artificial Intelligence and Evolutionary Computations in Engineering Systems. Advances in Intelligent Systems and Computing, 2020; 1056: 103–114. Springer, Singapore. doi: 10.1007/978-981-15-0199-9_9

[14] Milunovich G. Forecasting Australia’s real house price index: A comparison of time series and machine learning methods. Journal of Forecasting, 2020; 39(7): 1098–1118.

[15] Werbos PJ. Generalization of backpropagation with application to a recurrent gas market model, Neural Network. 1998; 1(4): 339–356.

[16] Li W, Luo Y, Zhu Q, et al. Applications of AR*-GRNN model for financial time series forecasting. Neural Computing and Applications 2008; 17: 441–448.

[17] Zhang D, Chen S, Ling L, Xia Q. Forecasting Agricultural Commodity Prices Using Model Selection Framework with Time Series Features and Forecast Horizons. IEEE Access 2020; 8: 28197–28209.

[18] Zhang GP, Qi M. Neural network forecasting for seasonal and trend time series. Eur. J. Oper. Res. 2005; 160: 501–514.

[19] Zhang GP, Kline DM. Quarterly time-series forecasting with neural networks. IEEE Trans. Neural Netw. 2007; 18: 1800–1814.

[20] Weng Y, Wang X, Hua J, Wang H, Kang M, Wang FY. Forecasting Horticultural Products Price Using ARIMA Model and Neural Network Based on a Large-Scale Data Set Collected by Web Crawler. IEEE Trans. Comput. Soc. Syst. 2019; 6: 547–553.

[21] Martínez F, Charte F, Frías MP et al. Strategies for time series forecasting with generalized regression neural networks, Neurocomputing, 2021: doi: 10.1016/j.neu]

[22] Liu Y, Duan Q, Wang D, Zhang Z, Liu C. Prediction for hog prices based on similar sub-series search and support vector regression, Comput. Electron. Agricult., 2019; 157: 581–588.

[23] Lu CJ, Lee TS, Chiu CC. Financial time series forecasting using independent component analysis and support vector machine. Decision Support Systems, 2009; 47(2): 115–125.

[24] Chen SY. Forecasting exchange rates: a new nonparametric support vector regression. The Journal of Quantitative & Technical Economics, 2007; 5: 142–150.

[25] Yu E, Wei H, Han Y et al. Application of time series prediction techniques for coastal bridge engineering. ABEN, 2021; 2, 6. doi: 10.1186/s43251-020-00025-4

[26] Douna V, Barraza V, Grings F, Huete A, et al. Towards a remote sensing data based evapotranspiration estimation in Northern Australia using a simple random forest approach, Journal of Arid Environment, 2021; 191: doi: 10.1016/j.jaridenv.2021.104513

[27] Jeong JH, Resop JP, Mueller ND, Fleisher DH, Yun K, Butler EE, et al. (2016) Random Forests for Global and Regional Crop Yield Predictions. PLoS ONE 11(6): e0156571. doi: 10.1371/journal.pone.0156571 27257967 PMC4892571

[28] Davis R, Nielsen M. Modeling of time series using random forests: Theoretical developments. Electronic Journal of Statistics, 2020; 14: 3644–3671.

[29] Sermpinis G, Stasinakis C, Theofilatos K, Karathanasopoulos A. Inflation and unemployment forecasting with genetic support vector regression. Journal of Forecasting, 2014; 33: 471–487.

[30] Paul RK, Vennila S, Singh N, Chandra P, Yadav SK, Sharma OP, et al. Seasonal Dynamics of Sterility Mosaic of Pigeonpea and its Prediction using Statistical Models for Banaskantha Region of Gujarat, India. Journal of The Indian Society of Agricultural Statistics, 2018; 72(3): 213–223.

[31] Breiman L. Random Forests. Machine Learning, 2001; 45: 5–32.

[32] Hillebrand E, Medeiros M. The benefits of bagging for forecast models of realized volatility. Econometric Reviews, 2010; 29: 571–593.

[33] Celikoglu HB. Application of radial basis function and generalized regression neural networks in non-linear utility function specification for travel mode choice modelling. Mathematical and Computer Modelling, 2006; 44(7–8): 640–658.

[34] Specht DF.A general regression neural network. IEEE Transactions on neural network, 1991; 2(6): 568–576. doi: 10.1109/72.97934 18282872

[35] Friedman JH. Greedy Function Approximation: A Gradient Boosting Machine. The Annals of Statistics, 2001; 29(5): 1189–1232.

[36] Friedman JH. Stochastic Gradient Boosting. Computational Statistics & Data Analysis, 2002; 38(4): 367–378.

[37] Diebold FX, Mariano RS. Comparing Predictive Accuracy. Journal of Business and Economic Statistics, 1995; 13: 253–63.

[38] Hansen PR, Lunde A, Nason JM. The model confidence set. Econometrica, 2011; 79(2): 453–497.

[39] Bernardi M, Catania L. The Model Confidence Set package for R. 2014; URL http://arxiv.org/abs/1410.8504

[40] Bokde ND, Yaseen ZM, Gorm BA. ForecastTB—An R Package as a Test-Bench for Time Series Forecasting—Application of Wind Speed and Solar Radiation Modeling. Energies, 2020; 13: 10: 2578.

